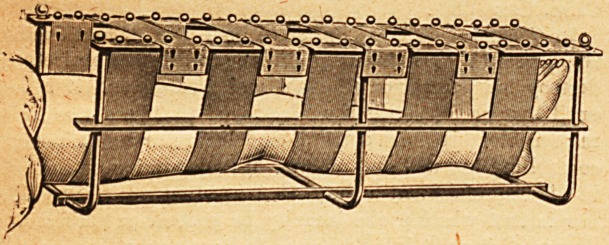# New Appliances and Things Medical

**Published:** 1907-02-16

**Authors:** 


### NEW APPLIANCES AND THINGS MEDICAL.

[We shall be glad to receive at our Office, 28 & 29 Southampton Street, Strand, London, W.C., from the manufacturers, specimens of all new preparations and appliances which may be brought out from time to time.]

### UNIVERSAL LEG SPLINT AND CRADLE.

(Jas. Woolley, Son and Co., Limited, Manchester.)

A useful *apparatus*, consisting of a combined leg splint and cradle, has been brought before our notice. A good idea of the splint may be gathered from the accompanying illustration. It consists of a galvanised metal frame 32 inches long, 7 inches deep, and 9 inches wide. Along the top on each side is fixed a row of brass studs, which serve to secure a series of ten straps. The straps are each 3 inches wide and are button-holed so as to be adjustable on the studs to half an inch. They are made of specially prepared waterproof material, and can be sterilised by boiling. The leg is suspended in these straps, and by adjusting these to different lengths of loops the leg can be placed in any position, with the knee flexed or extended and with the foot raised, depressed, or on a level with the thigh. By a careful adjustment of the straps equal support is given to the limb at all points. If alternate straps are buttoned tense across the top of the splint, the splint then acts as a cradle also, and removes all pressure of the bedclothes from the leg. An adjustable and detachable guard is provided also, in case the foot projects above the splint. Such an apparatus should prove useful in many cases of fracture and other injuries to the leg. The price is £1 10s.

### BANANINE BREAD AND BANANINE CREAM COCOA.

(Banana Bread, Flour, Food, Limited, 5 North John Street, Liverpool.)

We have received from the above Company a sample of bananine bread. This bread is made from bananine flour, the latter being derived from the unripe banana fruit. This banana flour, as compared with wheaten flour, contains more proteid, fat, and mineral matter, hence from the absolute nutritive standpoint banana flour or meal is more valuable than wheaten flour. If, then, we regard these two products from the money standard, much more nutritive material can be got for a given money unit from banana flour than from wheaten flour. In fact, from the economical standpoint we probably have in the banana one of the cheapest forms of nourishment extant. Banana flour or meal possesses certain properties which make it of interest to the medical practitioner, the chief one of which is the laxative action it possesses. This arises from its high content of mineral matter; it roughly contains five times the amount of mineral matter contained in wheaten flour. As we all know, brown bread or wholemeal bread has also a distinctly laxative action upon many individuals; this, however, is mostly due not to its mineral constituents, but to the fact that it contains an actual mechanical irritant in the shape of cellulose. This fact is worth noticing in this connection, because it is quite likely that bananine bread may be valuable for its special laxative properties to many patients unable to stand the mechanical irritation of wholemeal or brown bread. The sample of bananine bread which we have had the opportunity of examining is digestible and palatable. The bananine cream cocoa is a cocoa of good aroma, and makes an exceedingly pleasant drink. As was *a priori* to be expected, neither the bread nor the cocoa tastes appreciably of bananas, the compound ether to which this fruit owes its taste being volatile, and thus probably driven off in the drying process.

## Figures and Tables

**Figure f1:**